# Periodontal Pathogen Adhesion, Cytotoxicity, and Surface Free Energy of Different Materials for an Implant Prosthesis Screw Access Hole

**DOI:** 10.3390/medicina58020329

**Published:** 2022-02-21

**Authors:** Hsin-Ying Lu, Jason Hou, Yuta Takahashi, Yukihiko Tamura, Shohei Kasugai, Shinji Kuroda, Hidemi Nakata

**Affiliations:** 1 Department of Oral Implantology and Regenerative Dental Medicine, Tokyo Medical and Dental University, Tokyo 113-8510, Japan; hsinying.irm@tmd.ac.jp (H.-Y.L.); j_hou.irm@tmd.ac.jp (J.H.); kas.mfc@tmd.ac.jp (S.K.); 2 Dental Hospital Clinical Laboratory Division, Tokyo Medical and Dental University, Tokyo 113-8510, Japan; y57ydlab@tmd.ac.jp; 3 Department of Dental Pharmacology, Tokyo Medical and Dental University, Tokyo 113-8510, Japan; tamu.hpha@tmd.ac.jp

**Keywords:** dental implants, screw access hole materials, surface properties, biomaterials, peri-implantitis, bacteria adhesion, surface free energy, *Porphyromonas gingivalis*, *Fusobacterium nucleatum*, *Aggregatibacter actinomycetemcomitans*

## Abstract

*Background and Objectives*: Oral implant restorations are an excellent treatment option for edentulous patients; however, periodontopathogenic bacteria have been found in the microgaps between implant−abutment junctions. Implant designs to limit the microgaps have been extensively studied. However, studies have shown microgaps continue to exist, allowing for the leakage of bacteria into the implant system. Screw access hole materials are used to fill the access hole void. The use of materials with beneficial properties could provide bacterial leakage prevention. The aim of this study was to examine the surface free energy, cytotoxicity, and bacterial adhesion of selected screw access hole materials such as cotton, polytetrafluoroethylene (PTFE) tape, paraffin wax−polyolefin thermoplastic (PF), paraffin wax (Wax), gutta-percha (GP), and caviton EX (CE). *Materials and Methods*: A sessile drop test was performed to observe the contact angle and calculate the surface free energy of each material in order to determine the level of hydrophobicity. Cytotoxicity was examined in a mouse gingival epithelial cell line for day 1 and day 3. Bacterial adhesion was tested with *Porphyromonas gingivalis, Fusobacterium nucleatum,* and *Aggregatibacter actinomycetemcomitans*. *Results*: PTFE, PF, and wax presented low surface free energies of 19.34, 23.041, and 24.883 mN.m-1, respectively. No cytotoxicity was observed, except for GP and CE. Concurrently, the bacterial adhesion was also the lowest in PTFE and PF. *Conclusions*: Within the limits of this study, PTFE and PF showed an excellent biocompatibility with few bacterial adhesions. These materials could be potential screw access hole materials in clinical settings.

## 1. Introduction

In two-piece screw retained dental implant systems, it has been observed that progressive colonization by periodontopathogenic bacteria resides in the space between the implant components [1]. This space, known as the microgap, at the implant−abutment junction and screw access hole, provides a channel for bacterial colonization, which can lead to the accumulation of biofilm at the implant−abutment junction [1,2,3]. Ultimately, this can cause peri-implantitis, negatively affecting the short- and long-term outcomes of the treatment. Studies have found that significant proportions of periopathogens colonize the subgingival area of implants within 2–3 weeks from installation [4,5], with *Fusobacterium nucleatum* being the most established immediately after implantation [6]. *Prophyrogmonas gingivalis,* a bacterium linked to periodontitis [1,7,8], can attach to other oral bacterial species, such as *F. nucleatum* [7]. Additionally, cultures from individuals with peri-implantitis were shown to contain *Aggregatibacter actinomycetemcomitans* and *Prevotella intermedia* [9].

**Figure 1:**
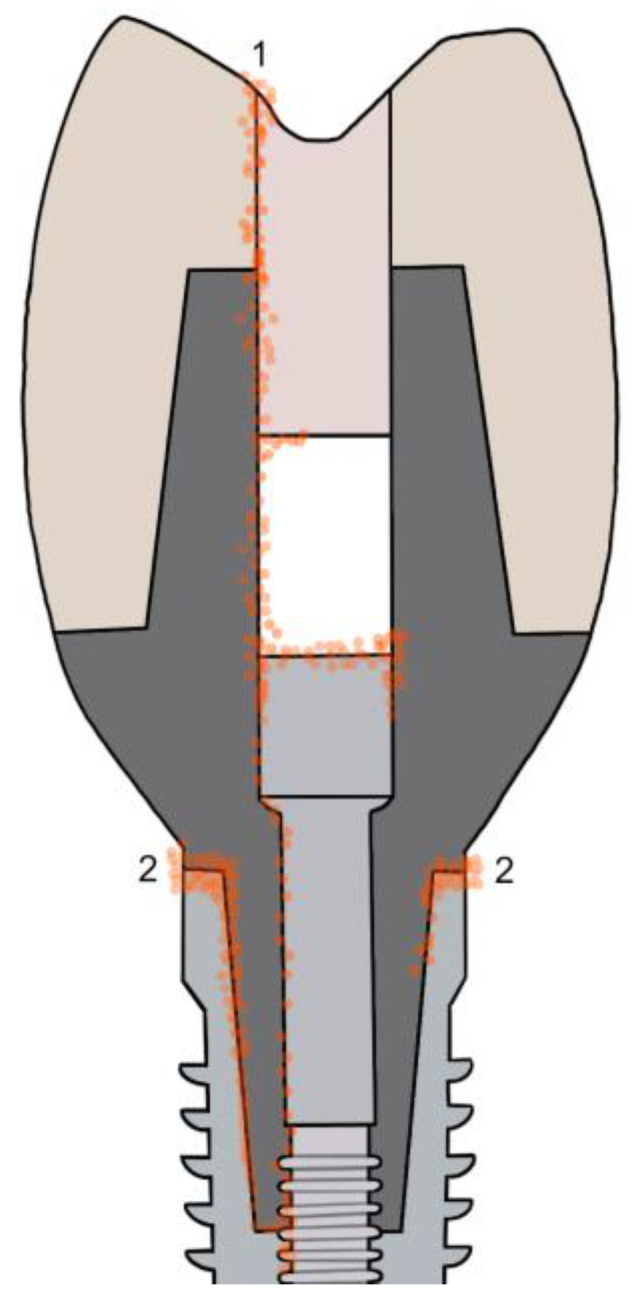
Possible leakage entry and exits of microorganism. (1) Occlusal screw access hole during sealing and/or occlusal function. (2) Implant−abutment junction microgap.

To combat peri-implantitis, the Morse taper design is utilized over the conventional dental implant system for its design for reducing the microorganism and inflammation surrounding the implant. The design is to reduce the microgap through high-precision intimate contact, and platform switching reduces marginal bone loss and provides additional space for tissue development [10]. However, the Morse taper does not eliminate the presence of a microgap. Studies have shown that Morse taper implant microgaps are still sufficient in size to be penetrated by oral bacteria [11]. Furthermore, despite the numerous implant systems, designs, and improvements, the existence of microgaps appears unavoidable [12]. Through the microgap, microorganism leakage can travel throughout the implant’s interior, eventually reaching the screw access hole. A common screw access hole material used in practice is cotton [13], but its non-ideal scaffold-like structure allows pathogenic bacteria to flourish, creating a harbor for growth. This is evident by visible blackening of the cotton associated with a strong malodor, primarily caused by Gram-negative oral bacteria [14,15]. The infiltration of microorganism could potentially create a source of bacteria, which then exits through the microgap, affecting the surrounding periodontium (Figure 1).

In other efforts to reduce peri-implantitis, platform switching is an alternative method to reduce the marginal bone loss. However, it may not be as important as the sealing of microgap leakage for determining peri-implant marginal bone level changes [16]. Addition to implant designs, the loosening of screws, introduction of bacteria during occlusal access hole sealing, and integrity of restorative material during occlusal function can be an entry for microorganism into the implant’s interior structures [2]. This shows that the implant’s interior is an important factor in implant treatment and should not be neglected. Unfortunately, none or very little effort has been put into the study of screw access hole materials against bacterial adhesion and surface energy.

The purpose of this study is to evaluate the cytotoxicity and bacterial adhesion of different materials used for screw access holes. The hypothesis of this study is that the testing materials will provide less susceptibility to bacterial adhesion. This study provides insight into the antibacterial properties and clinical safety of different materials; the surface free energy (SFE) was also examined to assess the wettability and hydrophobicity of the materials.

## 2. Materials and Methods

### 2.1. SFE Contact Angle Measurement

Cotton (Nikkosha, Tokyo, Japan), polytetrafluoroethylene tape (PTFE) (Kakudai, Osaka, Japan), paraffin wax–polyolefin thermoplastic tape (PF) (Bemis-Parafilm M, Neenah, WI, USA), paraffin wax (wax) (GC Corporation, Tokyo, Japan), gutta percha (GP) (GC Corporation, Tokyo, Japan), and Caviton EX (CE) (GC Corporation, Tokyo, Japan) were used as the substrate materials. Cotton was used as an additional control to observe any differences between the materials, while PTFE, PF, and wax allowed for easy manipulation and hydrophobic properties. All materials were commercially available. Furthermore, GP was selected for its availability and void-filing adaptability, and CE was selected for its good sealing properties as a restorative material with the potential to seal off the microgap when used as a screw access hole material.

**Figure 2:**
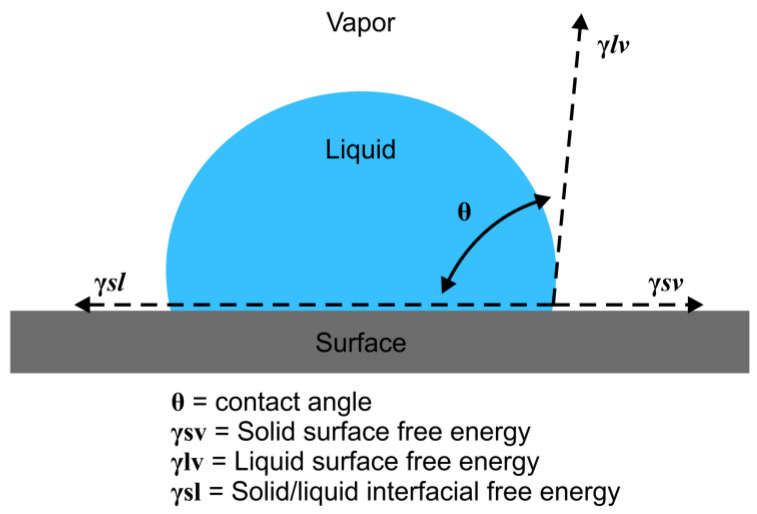
Schematic representation of Young’s equation.

PTFE, PF, and wax materials were prepared and cleaned with 70% ethanol and were air-dried. On the other hand, GP was heated and reshaped into a smooth flat disc, cleaned with 70% ethanol, and air-dried, while CE was used without any surface treatment. The sessile drop technique was utilized to measure the contact angles (Figure 2); each material was placed on a flat surface using a syringe with a 0.5 mm diameter needle, and a single drop of 0.2 mL of each liquid (water, ethanol (99.9%), glycerol, and dimethyl sulfoxide (DMSO)) was manually dispensed onto the substrate material with minimal impact. To achieve minimal impact, the tip of the needle was 1 mm away from the materials. Images of the static liquid drop were captured, and static contact angle measurements were performed using ImageJ software with a low-bond axisymmetric drop shape analysis (LBADSA) plugin [17,18]. The LBADSA was based on Young–Laplace equations, which are well adapted to drops on a horizontal substrate subject to gravity [19].

The mentioned liquids were selected due to their nontoxic properties and hence safe handling. Furthermore, the surface tension of the dispersive and polar components of the liquids has been demonstrated by Shen et al. [20] (Table 1).

#### SFE Calculations

Based on the contact angle measurements and dispersive and polar components of the liquids, the SFE of the materials was calculated using the linear form y=mx+b equation, as follows [17]:

(1) $$\left(\frac{\gamma_{l}\left(1+Cos\left(\theta \right)\right)}{2\sqrt{\gamma_{l}^{d}}}\right)=\sqrt{\gamma_{s}^{p}}\;\cdot \sqrt{\frac{\gamma_{l}^{p}}{\gamma_{l}^{d}}}+\sqrt{\gamma_{s}^{d}}$$

where $\gamma_{s}$ , $\gamma_{l}$ , and *θ* represent the SFE of the solid, SFE of the liquid, and contact angle, respectively. The superscripts *d* and *p* represent the dispersive and polar component, respectively.

Using this equation, four liquids with known dispersive and polar components for one solid surface were plotted. A linear trend line was developed with four data-points, and the linear equation was solved to determine *m* and *b*. Thus, the SFE of the solid was calculated using the following equation [17]:

(2) $$\gamma_{s}=m^{2}+b^{2}\;$$

This is based on the Owens and Wendt model, according to which SFE is the sum of two (polar and dispersive) contributions $\gamma_{s}=\gamma_{s}^{d}+\gamma_{s}^{p}\;$ [21].

### 2.2. Preparation of Materials

All six materials were prepared into a standardized size for the cell and bacterial assays. The materials were tightly fitted into a manufactured implant system consisting of an implant fixture, screw, and abutment (Intai Technology Corporation, Taichung, Taiwan). The dimensions of the implant were as follows: implant = Ø3.9 × 10 mm, abutment = Ø4.5, gingiva height 3 mm, and abutment height 5.5 mm. Each material was tightly sealed within the manufactured screw access hole to standardize the size of each test. Cotton and PTFE were sterilized in an autoclave at 121 °C and 115 kPa for 20 min. Other materials were disinfected with 75% ethanol due to their intolerability to the autoclave’s high temperature.

### 2.3. Cell Culture Preparation

A murine gingival epithelial cell line [22] (GE-1; (RCB1709) Riken Cell Bank, Tsukuba, Ibaraki, Japan) was used to test the toxicity of the materials. GE-1 cells were grown in a flask with serum free medium (SFM-101 medium) (Nissui, Tokyo, Japan) and 1% fetal bovine serum by incubating in a 5% CO_2_ atmosphere at 33°C for 96 h. The cells were unseeded and centrifuged at 1000 rpm for 3 min. After removal of the supernatant, the cells were resuspended in the same medium and subsequently the protocol of the EVE™ automated cell counter (NanoEntek, Waltham, MA, USA) was followed. Next, 10 μL of cells mixed with 0.4% Trypan Blue Solution were loaded onto an EVE™ slide and inserted into the EVE™ automated cell counter to calculate the desired concentration of 100,000 cells/mL. After this concentration was achieved, 1 mL of the GE-1 cells with a density of 100,000 cells/mL were seeded into a Corning HTS Transwell-24-well permeable supports (0.4 μm pore size for each well) and incubated for 48 h. The materials were then placed into the permeable supports of the Corning HTS transwell-24-well with no direct contact with the cells and sufficient submersion into the culture medium. The samples were incubated for 24 and 72 h before lactate dehydrogenase (LDH) and cell counting kit 8 (CCK8) assays were carried out.

### 2.4. LDH Assay

The levels of LDH, a stable enzyme in all the cells that release rapidly upon damage to the plasma membrane, were analyzed using a colorimetric assay. After the 24- and 72-h incubation periods (see above), 50 μL of solution from each well of the transwell 24-well plate was transferred into a 96-well plate followed by the addition of 50 μL of color reagent (LDH-Cytotoxic Test, FUJIFILM Wako Pure Chemical Corporation, Tokyo, Japan) and incubation for 40 min at room temperature (~25°C). Then, 100μL of stop solution LDH-Cytotoxic Test was added into each well and the absorbance of the samples was measured at 570 nm using an iMark Microplate Reader (Bio-Rad, Hercules, CA, USA).

### 2.5. Proliferation CCK8 Assay

The cytotoxicity of the materials to GE-1 cells was determined using the CCK8 kit assay (Dojindo, Kumamoto, Japan). After 24 and 72 h of incubation (see above), 45 μL of CCK8 solution was added to the transwell-24-well plates, where each well contained 450 μL of sample and was incubated at 33 °C for 60 min. Next, 100 μL of each reaction mixture was transferred onto a 96-well plate for the measurement of absorbance at 450/620 nm using an iMark Microplate Reader.

### 2.6. Bacterial Adhesion Assay

*P. gingivalis* (ATCC 33277), preserved in skim milk at −80°C, was obtained from the Department of Oral Implantology, Tokyo Medical and Dental University. *F. nucleatum* (ATCC 49256) and *A. actinomycetemcomitans* (ATCC 43717) were purchased from the American Type Culture Collection (ATCC, Manassas, VA, USA). *P. gingivalis* culture was grown in Vital Media Brucella HK Agar RS (Kyokuto Pharmaceutical Industrial Co., Ltd., Tokyo, Japan) by anaerobically incubating in an AnaeroPack System (Mitsubishi Gas Chemical, Tokyo, Japan) at 36 °C with 4.6% CO_2_ for 96 h. Anaerobic bacterial culture medium broth (Eiken Chemical Co., Ltd., Tokyo, Japan) was used to grow *P. gingivalis* broth culture and achieved an optical density with 600 nm (OD600) of 0.15–0.24 with a DU-64 spectrophotometer (Beckman Coulter, Brea, CA, USA).

To test bacterial adhesion, each substrate material was placed in bacterial cultures (1 mL) and incubated with the Anaeropack system for 96 h. At the indicated time-points, the samples were removed and briefly submerged in phosphate-buffered saline to remove any non-adherent bacteria on the surface of the material. These samples were serially diluted and the colony forming units (CFUs) were determined after 96 h of incubation.

*F. nucleatum* was grown similarly as *P. gingivalis* using the same growth media and incubation method. The OD600 of the *F. nucleatum* culture used for the assay was 0.15. *A. actinomycetemcomitans* was also cultured using a similar protocol; however, brain heart infusion agar and broth (Japan Becton Dickinson Co., Tokyo, Japan) were used. The organism was grown for 48 h in the AnaeroPack CO2 system and used at an OD600 of 0.15.

### 2.7. Statistical Analyses

Software SPSS (version 26.0; IBM, Armonk, NY, USA) and Prism 8.0 (GraphPad Software, La Jolla, CA, USA) were used to statistically analyze the obtained data. One-way ANOVA (post hoc multiple comparisons with Tukey’s test) tests were used to evaluate the overall significance and analyze comparisons. The data are presented as mean ± standard deviation and the results were considered statistically significant at *p* < 0.05.

## 3. Results

### 3.1. SFE

**Figure 3:**
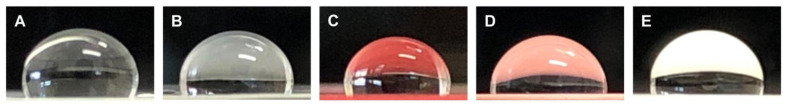
Image of the contact angle from a water sessile drop onto the materials. (**A**) PTFE, (**B**) paraffin, (**C**) wax, (**D**) gutta percha, and (**E**) caviton ex.

A sessile drop of water, ethanol, glycerol, or DMSO was preformed onto the surface of each material for a total of 25 measurements, as shown in Figure 3; the averages are shown in Table 2. Contact angle values near the complete wetting were considered to be 0° because of the difficulty in obtaining accurate values. Cotton was excluded from the analysis because of its complete wetting and complex fiber structure, and therefore no contact angles for the drops of the solutions were obtained. The degree of contact angles was used in the linear equation to obtain m and b, and the SFE of each material was determined using Equation (2) from Section 2.1.

**Figure 4:**
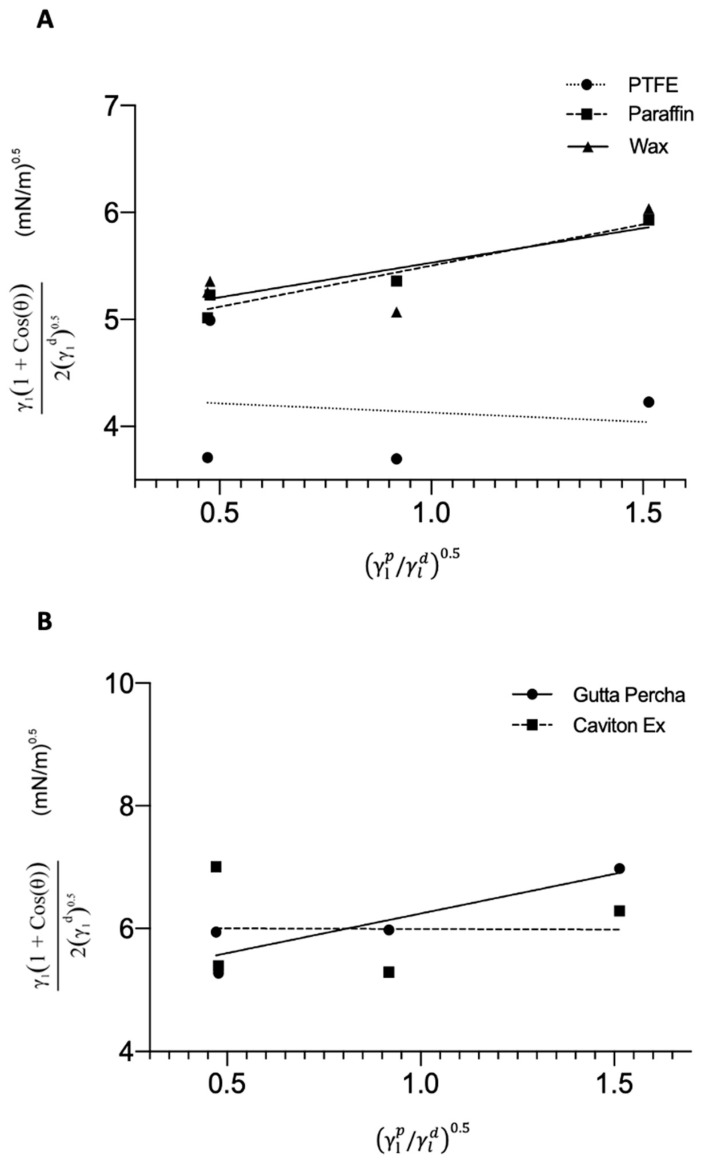
(**A**) Plot of the four liquids against PTFE, PF, and wax. The lines represent the best linear fit to the plotted point, respective to their material. The linear equation was used to solve for m and b to determine the SFE of the respective materials. (**B**) Plot of the four liquids against gutta percha and caviton ex. The lines represent the best linear fit to the plotted point, respective to their material. The linear equation was used to solve for m and b to determine the SFE of the respective materials.

Figure 4A,B shows the linear fitting parameters of m and b; 0.2637 and 4.3899, 0.7184 and 4.7461, 0.5286 and 4.9603, 1.1969 and 4.9819, and −0.1726 and 6.1487 for PTFE, PF, Wax, GP, and CE, respectively. PTFE showed the lowest SFE (19.34 mN.m-1) and the highest degree of contact angle among all of the other materials (Table 3), while CE was the most hydrophilic substrate among all the materials, with the highest SFE of 37.835 mN.m^−1^.

### 3.2. Cytotoxicity

**Figure 5:**
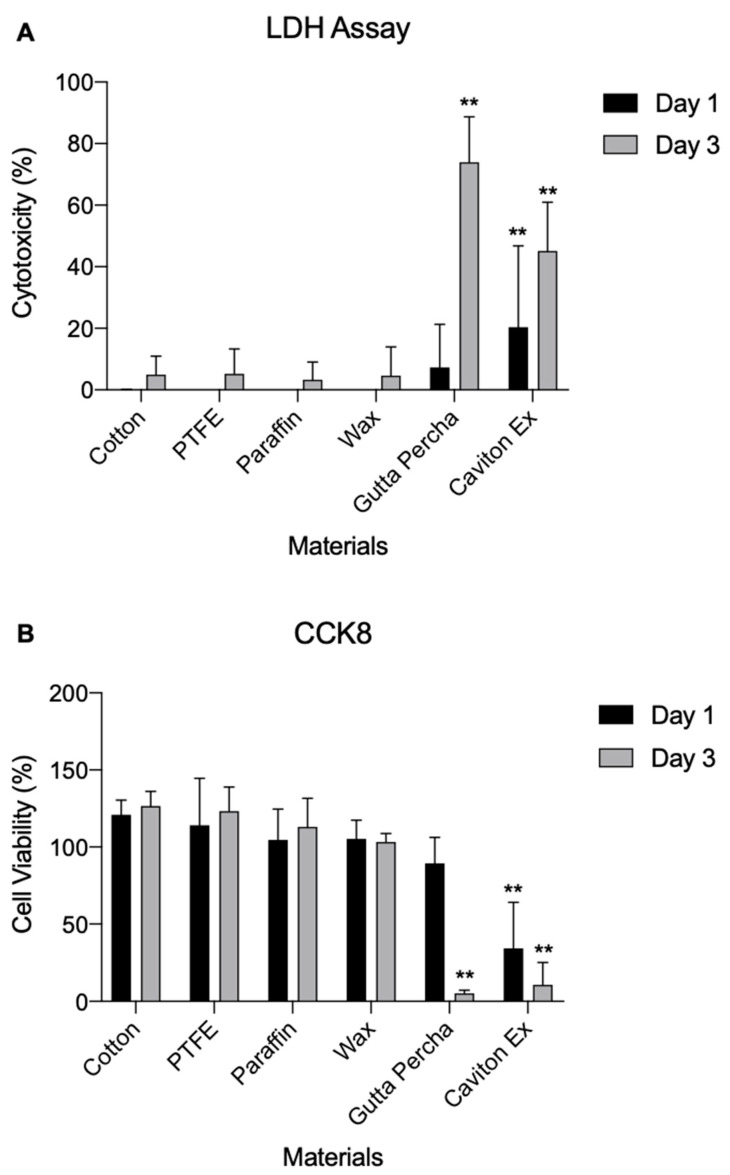
GE-1 cells cytotoxicity test. (**A**) Lactate dehydrogenase assay of the materials against GE-1 cells. The cells were incubated with the materials for 24 and 72 h. (**B**) Cell counting kit 8 assay of the materials. GE-1 cells were cultured for 24 and 72 h. Data are expressed as mean ± standard deviation (*n* = 10; ** *p* < 0.01 vs. cotton group).

The toxicity of each compound was tested against GE-1 cells. LDH and CCK8 assays were performed for 24 and 72 h, respectively. Among the six materials, both CE and GP showed a significant difference (*p* < 0.01) compared to cotton (Figure 5). On day 3, GP showed the highest level of LDH cytotoxicity, indicating toxicity to the GE-1 cells in comparison to cotton. PTFE, PF, and wax showed no signs of cytotoxicity compared to cotton.

**Figure 6:**
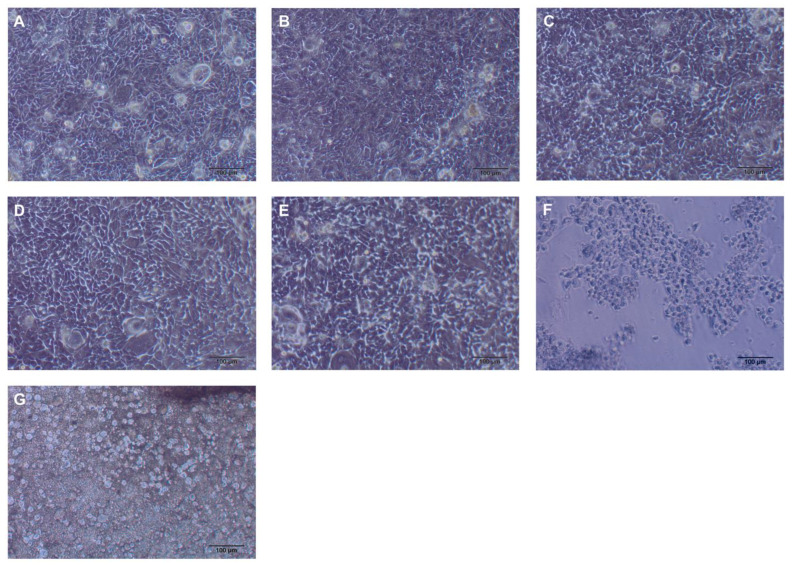
Day 1 samples of GE-1 cells incubated with materials under magnification 10×. (**A**) Control, (**B**) cotton, (**C**) PTFE, (**D**) paraffin, (**E**) wax, (**F**) gutta percha, and (**G**) caviton ex.

As shown in Figure 5B, the CCK8 assay results were consistent with that of the LDH assay. CE and GP showed the lowest cell viability of GE-1 cells, which was significantly lower than that of cotton and the other materials on day 3 (*p* < 0.01). The GE-1 cell samples were inspected under an Olympus IX70 Microscope with TH4-100 Lamp and DP25 photo image (Olympus, Tokyo, Japan) to observe the qualitative results (Figure 6).

### 3.3. CFU Counts

**Figure 7:**
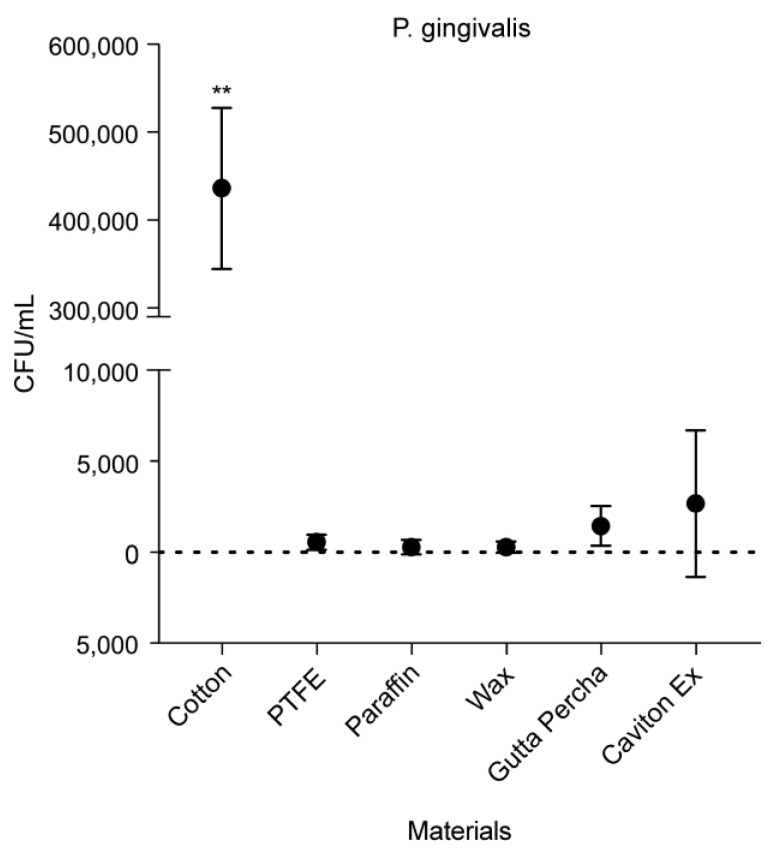
Colony forming units per mL of P. *gingivalis* adhesion against different materials. Data are expressed as mean ± standard deviation (*n* = 8; ** *p* < 0.01).

Both *P. gingivalis* and *F. nucleatum* were incubated with the materials for 92 h prior to counting, while *A. actinomycetemcomitans* was incubated for 48 h. As shown in Figure 7, cotton showed a significantly higher CFU count than all the other materials (*p* < 0.01), with an average count of 436,250 CFU/mL. Against *P. gingivalis*, PTFE, PF, and wax showed lower CFU counts of 520, 275, and 288 CFU/mL, respectively. On the other hand, GP (1450 CFU/mL) and CE (2675 CFU/mL) showed comparable counts.

**Figure 8:**
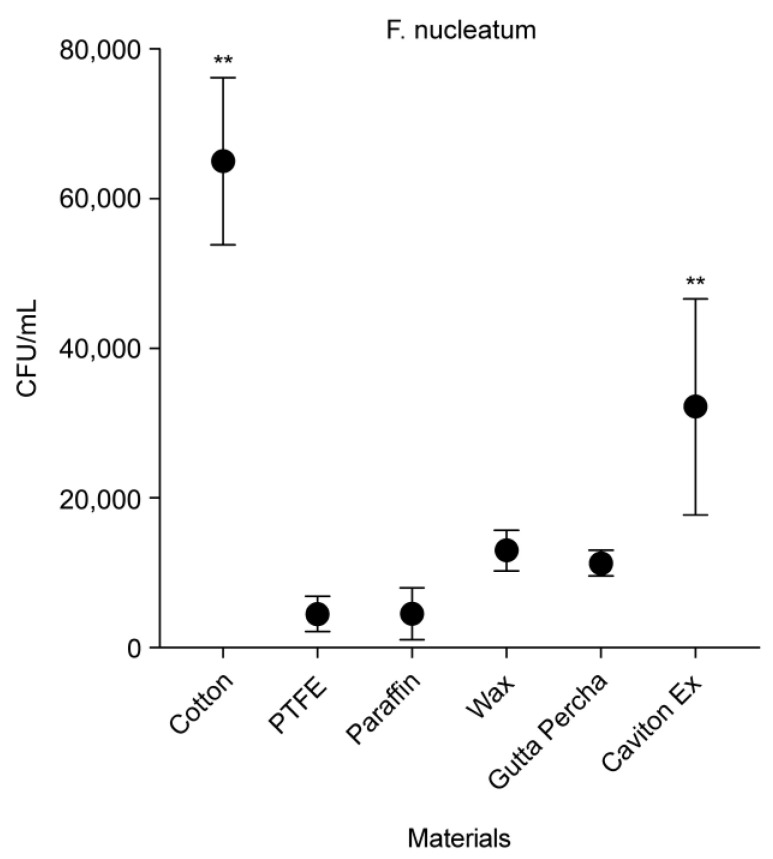
Colony forming units per mL of *F. nucleatum* adhesion against different materials. Data are expressed as mean ± standard deviation (*n* = 8; ** *p* < 0.01).

Similar to *P. gingivalis* adhesion, there were significant differences between the adhesion of cotton (average count of 65,025 CFU/mL; *p* < 0.01) and of all the other materials to *F. nucleatum* (Figure 8). PTFE, PF, wax, GP, and CE showed average *F. nucleatum* counts of 4513, 4575, 13,025, 11,325, and 32,225 CFU/mL, respectively. Evidently, CE’s *F. nucleatum* counts were significantly higher (*p* < 0.01) than that with all the other materials, except for cotton.

**Figure 9:**
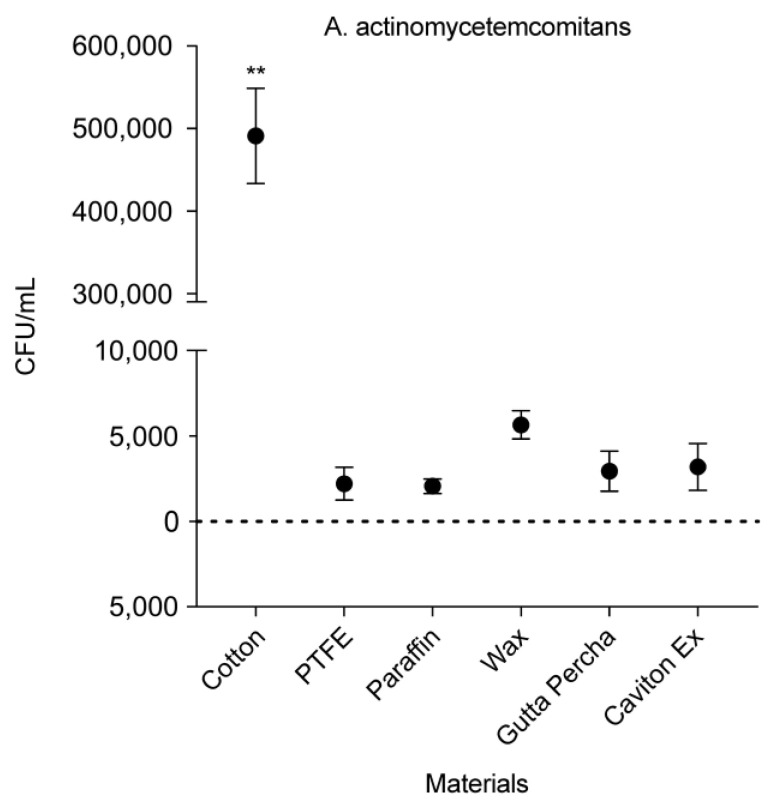
Colony forming units per mL of *A. actinomycetemcomitans* adhesion against different materials. Data are expressed as mean ± standard deviation (*n* = 8; ** *p* < 0.01).

As shown in Figure 9, cotton showed a significant level of *A. actinomycetemcomitans* adhesion (491,000 CFU/mL) in comparison to all the other materials (*p* < 0.01). Similar to the results with other bacterial strains, PTFE and PF showed the lowest *A. actinomycetemcomitans* densities.

## 4. Discussion

Most studies have focused on the implant exterior’s design and size to find a solution for bacterial contamination, while limited focus has been placed on the interior of the implant. The microgap facilitates potential bacterial leakage into and out of the internal structure of the implant system [23]. This is a common cause of inflammatory reactions at the marginal bone level, which can lead to ultimately marginal bone loss [24]. Thus, with the existence of the implant−abutment interface, screw access holes play an important role in the success of screw-retained implant treatment and should not be considered insignificant or neglected. 

The adhesion of bacteria to the implants is influenced by several factors, including physicochemical properties, such as the material’s surface, surface tension of the liquid, SFE of the material, and hydrophobicity. It is well known that the hydrophilicity and high SFE encourages the adhesion of bacteria to the materials [25]. Cotton pellets, which are rough surfaces with hydrophilic properties, have been reported to exhibit a significant increase in bacterial contamination [14]. In addition to its hydrophilic properties, the complex structure of porous materials provides shelter for bacteria [26]. Once bacteria are trapped inside the porous structure, they are difficult to remove from the cotton web [27].

The SFE technique described in this study is easy to perform and shows good reproducibility. The SFE of 19.34 (PTFE), 23.041 (PF), 24.883 (wax), and 26.251 mN.m-1 (GP) are considered low surface energies, which makes it difficult to bond, thus making them materials hydrophobic [28]. Additionally, Liu found that an SFE of 26 mN.m-1 and below showed excellent antimicrobial properties and reduced *Escherichia coli* adhesion [29]. This suggests that PTFE, PF, and wax could potentially reduce adhesion by other pathogenic bacteria. Di Giulio reported that a material with a contact angle measurement of 104° (against 0.9% NaCl; saline) showed significantly less adhesion with *P. gingivalis* [25]. In contrast with our study, water was one of the liquids used to measure the contact angles of the materials. Water has a lower surface tension of 72.75 mN.m-1 compared to NaCl (82 mN.m-1) and is more likely to spread across a surface than NaCl. PTFE and PF contact angles with water were 116° and 103.5°, respectively, showing that these two materials are much more hydrophobic and could potentially achieve significantly lower *P. gingivalis* adhesion.

The reduction in bacterial adhesion to the screw access hole of the material would greatly benefit the clinicians and the patients receiving the implant restoration. Considerable efforts have been made to evaluate different materials against three strains of Gram-negative bacteria that play important roles in peri-implantitis. The investigation showed that all the three strains adhered less to PTFE and PF. Van Dijk et al. reported that high SFE encourages bacterial adhesion [30]. Pereni et al. described that an SFE value of 25 mN.m-1 and lower is associated with minimal bacterial adhesion [31]. Consistently, we found that PTFE, PF, and wax were below 25 mN.m-1 and showed low CFU counts. Furthermore, PTFE and PF showed the lowest CFU counts of all the three strains of bacteria, with the PF-associated CFU counts being slightly lower. Although the SFE of GP and CE was higher than that of the other materials, they also showed significantly lower CFU counts than cotton. Based solely on the significantly low bacterial adhesion observed, PTFE, PF, wax, CE, and GP could be suggested as acceptable replacements for cotton. However, with biocompatibility, CE and GP are not ideal choices because of their observed cytotoxicity to GE-1 cells. The biocompatibility of implant materials is equally important in terms of antibacterial properties. GP and CE both showed a high toxicity towards gingival epithelial cells with indirect contact through the culture medium. This experimental model represents a clinical situation where the material has an indirect effect on the surrounding gingival cells through the saliva. GP contains zinc oxide, which may cause toxicity [32], most likely by diffusing out of the material when in contact with the fluids [33]. The results also demonstrated that the GP cytotoxicity was time-dependent (increased with time). CE also contains zinc oxide, explaining the associated cytotoxicity [34]. Cotton, PTFE, PF, and wax were found to be biocompatible with no cytotoxicity.

This assessment of bacterial adhesion to the implant materials is important and is linked to clinical performance. In particular, Gram-negative bacteria present in the screw access hole channel around the implant−abutment junction induce an inflammatory response near the bone level [35,36]. In contrast to in vitro studies, bacterial adhesion to any surface within the oral cavity is influenced by the acquired pellicle [37]. Bacterial adhesion in the oral cavity is a complicated process, and the findings of this study have limitations in clinical practice. However, the goal of this study was to distinguish screw access hole sealing materials in terms of bacterial adhesion, biocompatibility, and ease of accessibility in clinical settings.

## 5. Conclusions

This study showed that PTFE and PF materials were associated with significantly low CFU counts for all of the three test bacterial strains; PF had the lowest CFU count. Both of these materials could potentially be used as effective screw-hole materials. CE and GP materials also showed low bacterial adhesion, but were not viable as screw-access hole materials because of their observed cytotoxicity to GE-1 cells. The results of this study can help clinicians to select screw access hole materials other than cotton, possibly decreasing the risk of peri-implant disease.

## Figures and Tables

**Table 1:** Surface tension, and polar and dispersive components of liquids. Adapted with permission from [20]. Copyright 2022. American Chemical Society.

Solvent	Surface Tension	$\mathrm{Dispersive}\;\mathrm{Component}\;(\gamma^{d})$	$\mathrm{Polar}\;\mathrm{Component}\;(\gamma^{p})$
**Water**	72.75	22.10	50.65
**Ethanol**	23.70	19.30	4.40
**Glycerol**	63.40	29.00	34.40
**DMSO**	44.00	36.00	8.00

**Table 2:** Contact angle measurements of materials from the sessile liquid drop test (*n* = 25).

Material	Liquid			
	Water	Ethanol	Glycerol	Dimethyl Sulfoxide
PTFE	116.986° ± 5.02	31.748° ± 4.87	111.827° ± 2.50	89.3586° ± 4.76
Paraffin	103.492° ± 3.71	20.327° ± 1.80	95.125° ± 2.04	68.403° ± 4.35
Wax	102.710° ± 3.93	9.550° ± 1.48	97.960° ± 2.90	64.328° ± 4.30
Gutta Percha	95.634° ± 3.20	16.793° ± 0.92	89.113° ± 2.30	51.608° ± 3.56
Caviton Ex	100.801° ± 3.10	0° ± 0.0	95.795° ± 4.34	24.340° ± 9.28

**Table 3:** Surface free energy of the materials calculated from the data of the contact angle measurements.

	PTFE	Paraffin	Wax	Gutta Percha	Caviton Ex
SFE (mN.m^−1^)	19.34	23.041	24.883	26.251	37.835

## Data Availability

The data that support the findings of this study are available from the corresponding author upon reasonable request.

## References

[1] Cortelli S.C., Cortelli J.R., Romeiro R.L., Costa F.O., Aquino D.R., Orzechowski P.R., Araújo V.C., Duarte P.M. (2013). Frequency of periodontal pathogens in equivalent peri-implant and periodontal clinical statuses. Arch. Oral Biol..

[2] Alshehri M., Albaqiah H. (2017). Antimicrobial Efficacy of Materials Used for Sealing the Implant Abutment Screw Hole: An In Vitro Evaluation. Implant Dent..

[3] Canullo L., Radovanović S., Delibasic B., Blaya J.A., Penarrocha D., Rakic M. (2017). The predictive value of microbiological findings on teeth, internal and external implant portions in clinical decision making. Clin. Oral Implants Res..

[4] Teughels W., Van Assche N., Sliepen I., Quirynen M. (2006). Effect of material characteristics and/or surface topography on biofilm development. Clin. Oral Implants Res..

[5] Fürst M.M., Salvi G.E., Lang N.P., Persson G.R. (2007). Bacterial colonization immediately after installation on oral titanium implants. Clin. Oral Implants Res..

[6] van Winkelhoff A.J., Goené R.J., Benschop C., Folmer T. (2000). Early colonization of dental implants by putative periodontal pathogens in partially edentulous patients. Clin. Oral Implants Res..

[7] Park Y., Simionato M.R., Sekiya K., Murakami Y., James D., Chen W., Hackett M., Yoshimura F., Demuth D.R., Lamont R.J. (2005). Short fimbriae of Porphyromonas gingivalis and their role in coadhesion with Streptococcus gordonii. Infect. Immun..

[8] Persson G.R., Renvert S. (2014). Cluster of bacteria associated with peri-implantitis. Clin. Implant Dent. Relat. Res..

[9] Leonhardt A., Dahlén G., Renvert S. (2003). Five-year clinical, microbiological, and radiological outcome following treatment of peri-implantitis in man. J. Periodontol..

[10] Macedo J.P., Pereira J., Vahey B.R., Henriques B., Benfatti C.A.M., Magini R.S., López-López J., Souza J.C.M. (2016). Morse taper dental implants and platform switching: The new paradigm in oral implantology. Eur. J. Dent..

[11] Ranieri R., Ferreira A., Souza E., Arcoverde J., Dametto F., Gade-Neto C., Seabra F., Sarmento C. (2015). The bacterial sealing capacity of morse taper implant-abutment systems in vitro. J. Periodontol..

[12] Lopes de Chaves E., Mello Dias E.C., Sperandio M., Napimoga M.H. (2018). Association between Implant-Abutment Microgap and Implant Circularity to Bacterial Leakage: An In Vitro Study Using Tapered Connection Implants. Int. J. Oral Maxillofac. Implants.

[13] Tarica D.Y., Alvarado V.M., Truong S.T. (2010). Survey of United States dental schools on cementation protocols for implant crown restorations. J. Prosthet. Dent..

[14] Raab P., Alamanos C., Hahnel S., Papavasileiou D., Behr M., Rosentritt M. (2017). Dental materials and their performance for the management of screw access channels in implant-supported restorations. Dent. Mater. J..

[15] Schoenbaum T.R., Wadhwani C., Stevenson R.G. (2017). Covering the Implant Prosthesis Screw Access Hole: A Biological Approach to Material Selection and Technique. J. Oral Implantol..

[16] Wang Y.C., Kan J.Y., Rungcharassaeng K., Roe P., Lozada J.L. (2015). Marginal bone response of implants with platform switching and non-platform switching abutments in posterior healed sites: A 1-year prospective study. Clin. Oral Implants Res..

[17] Vicente C.M.S., André P.S., Ferreira R.A.S. (2012). Simple measurement of surface free energy using a web cam. Rev. Bras. Ensino Fis..

[18] Di Giulio M., Traini T., Sinjari B., Nostro A., Caputi S., Cellini L. (2016). Porphyromonas gingivalis biofilm formation in different titanium surfaces, an in vitro study. Clin. Oral Implants Res..

[19] Stalder A.F., Melchior T., Müller M., Sage D., Blu T., Unser M. (2010). Low-bond axisymmetric drop shape analysis for surface tension and contact angle measurements of sessile drops. Colloids Surf. A Physicochem. Eng. Asp..

[20] Shen J., He Y., Wu J., Gao C., Keyshar K., Zhang X. (2015). Liquid Phase Exfoliation of Two-Dimensional Materials. Nano Lett..

[21] Owens D.K., Wendt R.C. (1969). Estimation of the surface free energy of polymers. J. Appl. Polym. Sci..

[22] Hatakeyama S., Ohara-Nemoto Y., Yanai N., Obinata M., Hayashi S., Satoh M. (2001). Establishment of gingival epithelial cell lines from transgenic mice harboring temperature sensitive simian virus 40 large T-antigen gene. J. Oral Pathol. Med..

[23] Teixeira W., Ribeiro R.F., Sato S., Pedrazzi V. (2011). Microleakage into and from two-stage implants: An in vitro comparative study. Int. J. Oral Maxillofac. Implants.

[24] Berberi A., Maroun D., Kanj W., Amine E.Z., Philippe A. (2016). Micromovement Evaluation of Original and Compatible Abutments at the Implant-abutment. Interface J. Contemp. Dent. Pract..

[25] van Loosdrecht M.C., Lyklema J., Norde W., Schraa G., Zehnder A.J. (1987). The role of bacterial cell wall hydrophobicity in adhesion. Appl. Environ. Microbiol..

[26] Varshney S., Sain A., Gupta D., Sharma S. (2021). Factors Affecting Bacterial Adhesion on Selected Textile Fibres. Indian J. Microbiol..

[27] Hemmatian T., Lee H., Kim J. (2021). Bacteria Adhesion of Textiles Influenced by Wettability and Pore Characteristics of Fibrous Substrates. Polymers.

[28] Categorizing Surface Energy. 3M. Access 2022. https://www.3m.co.id/3M/en_ID/bonding-and-assembly-id/resources/science-of-adhesion/categorizing-surface-energy/.

[29] Liu Y., Zhao Q. (2005). Influence of surface energy of modified surfaces on bacterial adhesion. Biophys. Chem..

[30] van Dijk J., Herkströter F., Busscher H., Weerkamp A., Jansen H., Arends J. (1987). Surface-free energy and bacterial adhesion. An in vivo study in beagle dogs. J. Clin. Periodontol..

[31] Pereni C.I., Zhao Q., Liu Y., Abel E. (2006). Surface free energy effect on bacterial retention. Colloids Surf. B Biointerfaces.

[32] Szep S., Grumann L., Ronge K., Schriever A., Schultze M., Heidemann D. (2003). In vitro cytotoxicity of medicated and nonmedicated gutta-percha points in cultures of gingival fibroblasts. J. Endod..

[33] Pascon E.A., Spngberg L.S.W. (1990). In vitro cytotoxicity of root canal filling materials: 1. Gutta-percha. J. Endod..

[34] Prabhakar A.R., Shantha Rani N., VNaik S. (2017). Comparative Evaluation of Sealing Ability, Water Absorption, and Solubility of Three Temporary Restorative Materials: An in vitro Study. Int. J. Clin. Pediatr. Dent..

[35] Broggini N., McManus L.M., Hermann J.S., Medina R.U., Oates T.W., Schenk R.K., Buser D., Mellonig J.T., Cochran D.L. (2003). Persistent acute inflammation at the implant-abutment interface. J. Dent. Res..

[36] do Nascimento C., Barbosa R.E., Issa J.P., Watanabe E., Ito I.Y., Albuquerque R.F. (2008). Bacterial leakage along the implant-abutment interface of premachined or cast components. Int. J. Oral Maxillofac. Surg..

[37] Whittaker C.J., Klier C.M., Kolenbrander P.E. (1996). Mechanisms of adhesion by oral bacteria. Annu. Rev. Microbiol..

