# Correction: Lu et al. Periodontal Pathogen Adhesion, Cytotoxicity, and Surface Free Energy of Different Materials for an Implant Prosthesis Screw Access Hole. *Medicina* 2022, *58*, 329

**DOI:** 10.3390/medicina58101413

**Published:** 2022-10-09

**Authors:** Hsin-Ying Lu, Jason Hou, Yuta Takahashi, Yukihiko Tamura, Shohei Kasugai, Shinji Kuroda, Hidemi Nakata

**Affiliations:** 1 Department of Oral Implantology and Regenerative Dental Medicine, Tokyo Medical and Dental University, Tokyo 113-8510, Japan; 2 Dental Hospital Clinical Laboratory Division, Tokyo Medical and Dental University, Tokyo 113-8510, Japan; 3 Department of Dental Pharmacology, Tokyo Medical and Dental University, Tokyo 113-8510, Japan

## Error in Figure

In the original publication [1], there were some mistakes in Figures 4 and 5 as published. In Figure 4, the *X*-axis is corrected, but the label of the *X*-axis was missing a “/” symbol, The corrected Figure 4 appears below.

In Figure 5 GE-1 cells cytotoxicity test, there was statistical mistake as published. The corrected Figure 5 appears below.

## Text Correction

There was an error in the original publication due to the statistical mistake. A correction has been made to ***Section 3.2. Cytotoxicity***. The corrected paragraph appears below.

The toxicity of each compound was tested against GE-1 cells. LDH and CCK8 assays were performed for 24 and 72 h, respectively. Among the six materials, both CE and GP showed a significant difference (*p* < 0.01) compared to cotton (Figure 5). On day 3, GP showed the highest level of LDH cytotoxicity, indicating toxicity to the GE-1 cells in comparison to cotton. PTFE, PF, and wax showed no signs of cytotoxicity compared to cotton.

As shown in Figure 5B, the CCK8 assay results were consistent with that of the LDH assay. CE and GP showed the lowest cell viability of GE-1 cells, which was significantly lower than that of cotton and the other materials on day 3 (*p* < 0.01). The GE-1 cell samples were inspected under an Olympus IX70 Microscope with TH4-100 Lamp and DP25 photo image (Olympus, Tokyo, Japan) to observe the qualitative results (Figure 6).

This correction was approved by the Academic Editor. The original publication has also been updated.

## Figures and Tables

**Figure 4:**
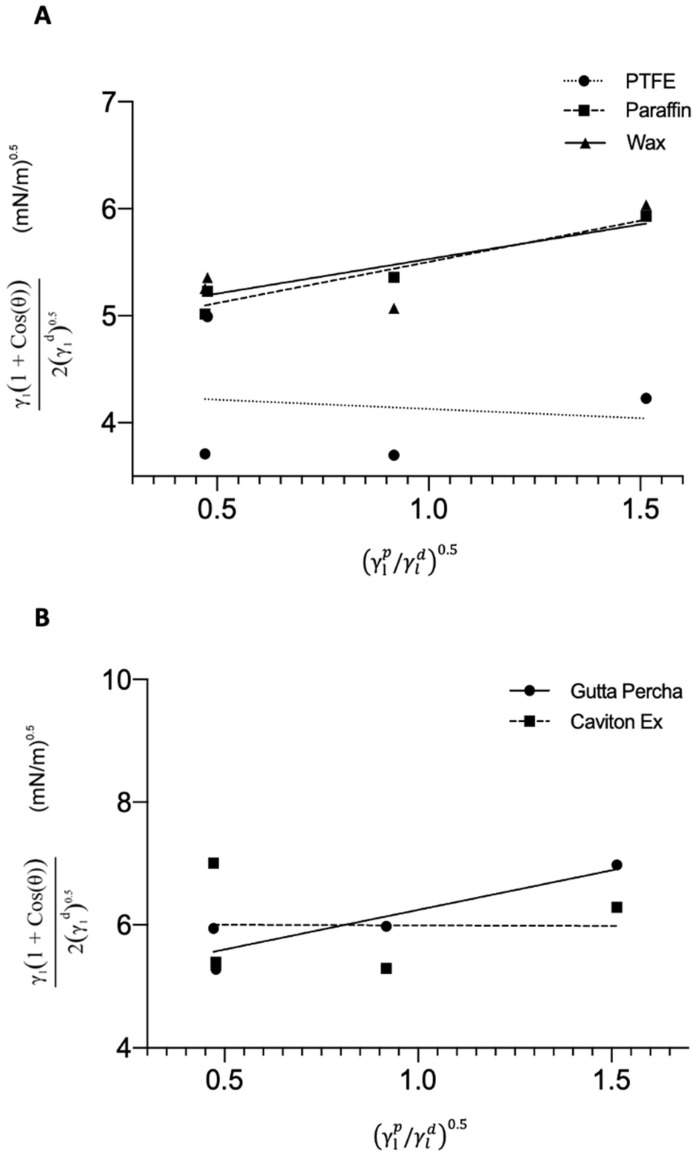
(**A**) Plot of the four liquids against PTFE, PF, and wax. The lines represent the best linear fit to the plotted point, respective to their material. The linear equation was used to solve for m and b to determine the SFE of the respective materials. (**B**) Plot of the four liquids against gutta percha and caviton ex. The lines represent the best linear fit to the plotted point, respective to their material. The linear equation was used to solve for m and b to determine the SFE of the respective materials.

**Figure 5:**
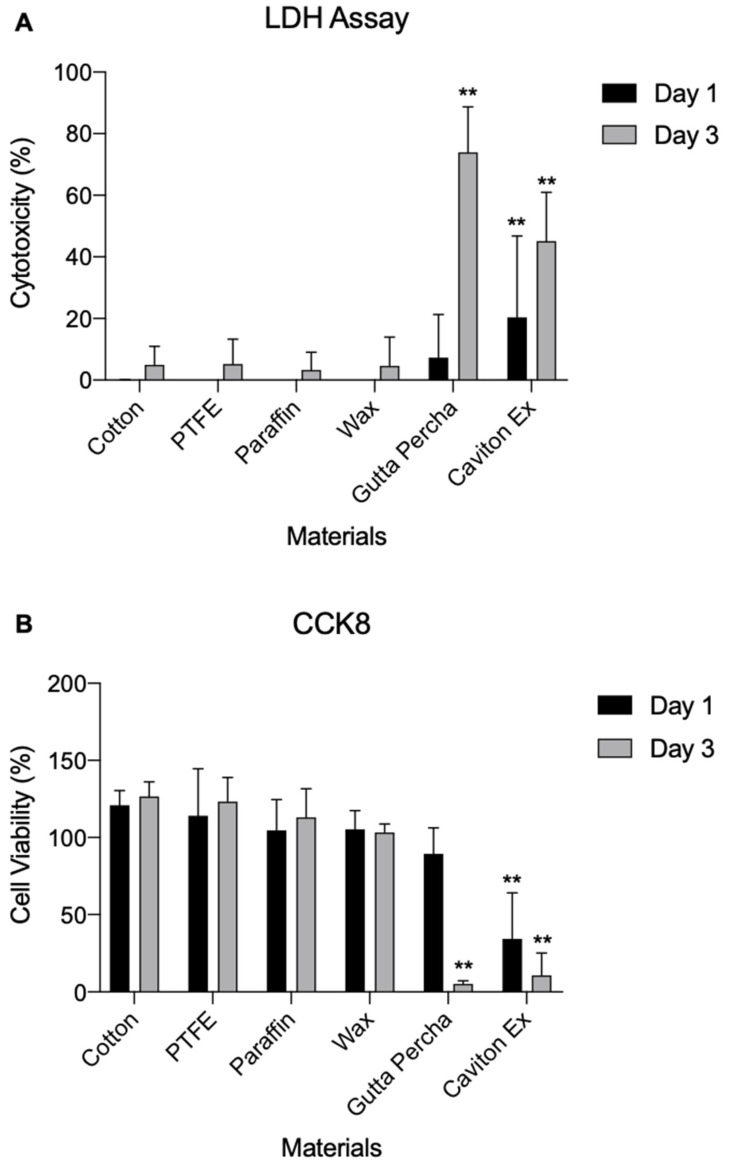
GE-1 cells cytotoxicity test. (**A**) Lactate dehydrogenase assay of the materials against GE-1 cells. The cells were incubated with the materials for 24 and 72 h. (**B**) Cell counting kit 8 assay of the materials. GE-1 cells were cultured for 24 and 72 h. Data are expressed as mean ± standard deviation (*n* = 10; ** *p* < 0.01 vs. cotton group).
